# Impact of Platelet-Rich Plasma on the Rate of Canine Retraction: A Split-Mouth Randomized Clinical Trial

**DOI:** 10.7759/cureus.108139

**Published:** 2026-05-02

**Authors:** Neharika Awod, Archana Jatania, Balaji Kendre, Amit Akolkar, Mandar Pathak, Gopal Chavan

**Affiliations:** 1 Orthodontics, Saraswati Dhanwantari Dental College and Hospital, and Post Graduate and Research Institute, Parbhani, IND

**Keywords:** accelerated tooth movement, canine retraction, pharmacological approach, platelet-rich plasma (prp), retraction

## Abstract

Background: The main concern of patients undergoing orthodontic treatment is the prolonged duration of treatment. Various measures have been introduced in the field of orthodontics to accelerate tooth movement. Tooth acceleration can be carried out by pharmacological and surgical methods; among them, platelet-rich plasma (PRP) is a minimally used method that has beneficial effects on skin, bone, and musculature. To understand its effect on the rate of tooth movement, this study aims to evaluate the effect of PRP therapy on the retraction rate in a split-mouth study design.

Materials and methods: A split-mouth study was conducted on 30 patients requiring bilateral extraction of upper first premolars. PRP was prepared and injected into the alveolar mucosa, distal to the canine on experimental sides, with the contralateral sides acting as a control side. Canine retraction was started on the same day of PRP injection. Retraction was started using NiTi closed coil springs on both sides. The amount of canine retraction was measured and compared between both sides on 0 day, 21 days, and 42 days.

Results: The results showed the mean rate of canine retraction in group PRP was 5.66±0.51, 4.50±0.54, and 3.16±0.40, respectively, at 0 day, 21 days, and 42 days, with a statistically significant difference between the control group (p<0.001). Increased tooth movement was observed on the experimental side compared to the control group.

Conclusion: PRP injections are a minimally invasive procedure and a safe approach for accelerating canine retraction and reducing overall treatment time. PRP can be used as an adjunct for accelerating the orthodontic tooth movement.

## Introduction

Orthodontic tooth movement results from a biological reaction to a force applied or derived to the dentofacial or orofacial region that interferes with physiological homeostasis [1]. The main concern of patients undergoing active orthodontic treatment is the prolonged duration of treatment, varying between 2 and 3 years, depending on the individual aspect of severity in patients. Therefore, various measures have been introduced in the field of orthodontics that can reduce the duration of the treatment time and bring about accelerated tooth movement. Various approaches, such as pharmacological, physical [2,3], and surgical [4,5] methods, have been introduced to accelerate tooth movement [6,7].

Pharmacological approaches include administration of local or systemic biological factors, despite adverse reactions such as local pain and root resorption having been reported [8]. Similar to pharmacological approaches, mechanical and physical approaches have also been used, which utilize various lasers to physical stimulators like vibrational frequency that brings about acceleration [9-12]. Surgical interventions such as corticotomy and piezocision have also been reported for accelerated orthodontics [13,14]. Though the surgical intervention has been proven to be the most effective one currently, its disadvantages are that it’s an ostectomy procedure, which causes loss of alveolar bone architecture that undermines the periodontal support of the target teeth [15]. All the procedures for accelerated orthodontics undergo a regional acceleratory phenomenon to bring about the acceleration [16]. They involve cellular and chemical changes that increase the osteoclastic activity via RANKL/RANK that further helps in bone remodeling, resulting in increased tooth movement in a shorter period of time [15].

There has been a surge to investigate various methods that bring about acceleration of orthodontic tooth movement. Recent advances in accelerated orthodontics include a novel, minimally invasive technique including administration of local agents such as platelet-rich plasma [8] (PRP) [17]. PRP is an autologous concentration of platelets in a small volume of plasma [17]. The growth factors within the PRP have the potential to stimulate osteoblastic and osteoclastic activities, together with their rich content of cytokines, which have a major role during tooth movement [18-21]. PRP was employed in conjunction with autogenous bone grafts for mandibular deformity restoration. According to radiography, PRP with bone grafts showed a greater bone density and maturation rate than bone grafts.

Throughout mediating differentiation, activation and survival of all the bone cells supported the idea that PRP could have a possible effect on orthodontic tooth movement and can be used for en masse retraction tooth movement [22]. There isn't much information on the many applications of PRP in orthodontics [8, 22], despite the fact that it has been utilized to treat periodontal defects [23], extraction socket repair, sinus lift augmentation, and periapical osseous defects, among other conditions. Al‑Bozaie et al. [22] reported clinical outcomes of PRP injections during en masse retraction, providing a directly comparable clinical context. While contrasting PRP with other platelet concentrates, Ammar et al. [8] compared PRP and i‑PRF in a randomized trial, highlighting differences in preparation and clinical effect sizes. PRP can be used as an emerging adjunct for accelerating tooth movement, as shown by recent clinical use of PRP in orthodontic retraction [22]. Hence, the present study aims to evaluate the effect of plasma-rich protein therapy on the retraction rate of human maxillary canines at different intervals of time such as 21st day and 42nd day and also as the 21-day versus 42-day intervals in a split-mouth study design. The primary outcome was the amount of canine retraction (mm) at 42 days; the trial tested the superiority of PRP versus control with a clinically meaningful difference set at X mm. The null hypothesis was that there would be no statistically significant difference in the amount of canine retraction between the PRP and control sides at the defined timepoints.

Limited split-mouth randomized controlled trials with standardized PRP protocols and short-term biologic assessment intervals are available. The present study aimed to address these methodological inconsistencies and provide standardized clinical evidence.

Clinical implications

The application of PRP demonstrates a promising role in accelerating orthodontic tooth movement, particularly during canine retraction. By enhancing local bone remodeling through the release of growth factors, PRP facilitates increased osteoclastic and osteoblastic activity, leading to faster tooth displacement. Clinically, this can contribute to a reduction in the duration of space closure phases, thereby shortening overall treatment time. Additionally, its autologous nature ensures biocompatibility with minimal adverse effects, making PRP a viable adjunct in routine orthodontic practice aimed at improving treatment efficiency and patient compliance

## Materials and methods

The study was designed to be a split-mouth randomized controlled trial. This clinical trial was conducted among 30 patients from the Department of Orthodontics and Dentofacial Orthopaedics undergoing fixed orthodontic treatment. The study was registered with the Clinical Trial Registration number CTRI/2024/04/066522. Institutional Ethical approval was obtained with the ethical clearance number SDDCH./Admin/695/2023. 

The inclusion criteria consisted of patients aged between 17 and 35 years with skeletal class I jaw base with normodivergent growth pattern and Angle's class I Dewey's type 2 malocclusion patients. Patients with crowding with little irregularity index from 0 to 2 and proclination greater than 5mm were selected, and those requiring extraction of 1st premolar as part of orthodontic treatment with good general and oral health without any history of drug usage or any skeletal or dental deformity, patients with a normal bilateral chewing pattern, and patients with a mixed diet (balanced diet) were included.

 The exclusion criteria included individuals with a history of long‑term medication because nonsteroidal anti‑inflammatory drugs and hormone supplements are known to interfere with bone metabolism, individuals with unilateral chewing or parafunctional habits, skeletal crossbite, and occlusal interferences. Patients selected for this clinical trial were informed about the entire procedure and informed consent was obtained from each patient. Confounding variables were minimized by adopting a split-mouth design, wherein each subject served as their own control, thereby reducing inter-individual variability. Additionally, random allocation of intervention sides, standardization of orthodontic force application, and consistent measurement protocols were employed to control residual confounding factors.

Sample size calculation

The values of mean canine retraction in both groups from the study conducted by Ammar et al. (2018) [8] were substituted in OpenEpi Version 3, an open-source calculator, and the sample size was calculated as follows:

\[n_{1} = \frac{\left( \sigma^{2} + \frac{\sigma^{2}}{\kappa} \right)\left( Z_{1-\alpha/2} + Z_{1-\beta} \right)^{2}} {\Delta^{2}}\]

\[n_{2} = \frac{\left( \kappa \sigma^{2} + \sigma^{2} \right)\left( Z_{1-\alpha/2} + Z_{1-\beta} \right)^{2}} {\Delta^{2}} \]

The notation for the formulae is: n1 = sample size of Group 1; n2 = sample size of Group 2; Ϭ1 = standard deviation of Group 1=1.02; Ϭ2 = standard deviation of Group 2=1.01; Δ = difference in group means=1.12; к = ratio = n1/ n2=1; Z1-α/2 = two-sided Z value (e.g., Z=1.96 for 95% confidence interval); Z1-β = power =95%; sample size= 22 each group

Corrected sample size assuming 25% attrition due to error,

= 22/0.75=29.33, which was rounded off to 29.30

**Figure 1:**
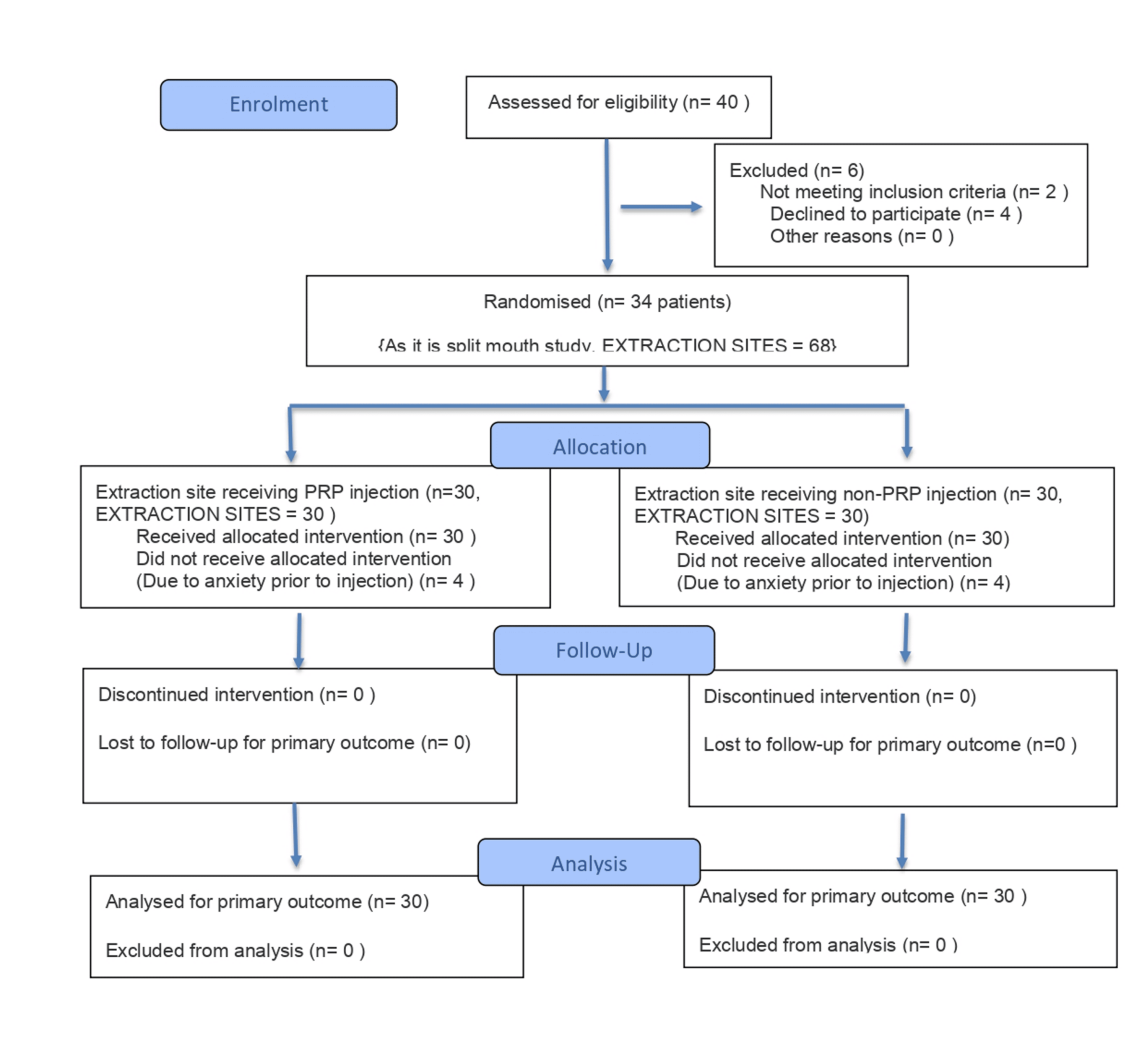
CONSORT diagram. CONSORT: Consolidated Standards of Reporting Trials

There is a total of two groups. Therefore, the total sample size is 30×2= 60 samples. Since this is a split-mouth study, 60 sites will be required in 30 patients [23], as depicted in Figure 1.

Sample selection and randomization

Sample selection was done by the convenience method by selecting the patient based on the inclusion criteria. Participants were initially recruited using a convenience sampling approach from patients reporting to the Department of Orthodontics who met the predefined inclusion and exclusion criteria. After eligibility confirmation and informed consent, a simple randomization method was used to allocate the experimental and control sides in the split-mouth design. 

Sample demographics

The sample demographics were Angle's class I malocclusion with Dewey's type 2 modification cases requiring first premolar bilateral extraction. The skeletal demographics were skeletal class I jaw base.

Allocation concealment

Allocation concealment was performed using sequentially numbered, opaque, sealed envelopes prepared by an independent investigator who was not involved in outcome assessment. The envelope was opened only at the time of intervention. 

Methodology

Full preoperative records were obtained and MBT(0.022”) brackets were bonded. Levelling and alignment were carried out until the 0.019X0.025-inch stainless steel archwire was reached. Study impressions were taken before the start and after the end of the retraction procedure. In each patient, a quadrant of the maxilla to be injected with PRP was selected randomly, while the contralateral side was considered the control side to eliminate the risk of bias in the study.

Platelet-rich plasma preparation

Venous blood samples were collected from the pre-cubital vein of each patient in vacutainers containing EDTA as an anticoagulant and mixed well. The blood was used for preparation of PRP by the method of double spin centrifugation described by Marx and Garg [8], immediately after blood sample collection. The first spin was performed at 1500 rpm for 10 min, from which three layers were formed: the upper layer of platelets and white blood cells, the middle buffy coat layer, and the bottom red blood cells layer. The top layer and middle buffy coat layer were carefully separated into another sterile test tube and centrifuged again at 2500 rpm for another 10 min, giving PRP. The platelet concentration achieved after double-spin centrifugation was approximately 3.47-5 times baseline whole blood values. The obtained PRP was collected in a 30G Insulin syringe for injecting into the recipient site. PRP platelet concentration was measured and averaged X ± Y platelets/µL; PRP was activated prior to injection

Injection of platelet-rich plasma

**Figure 2:**
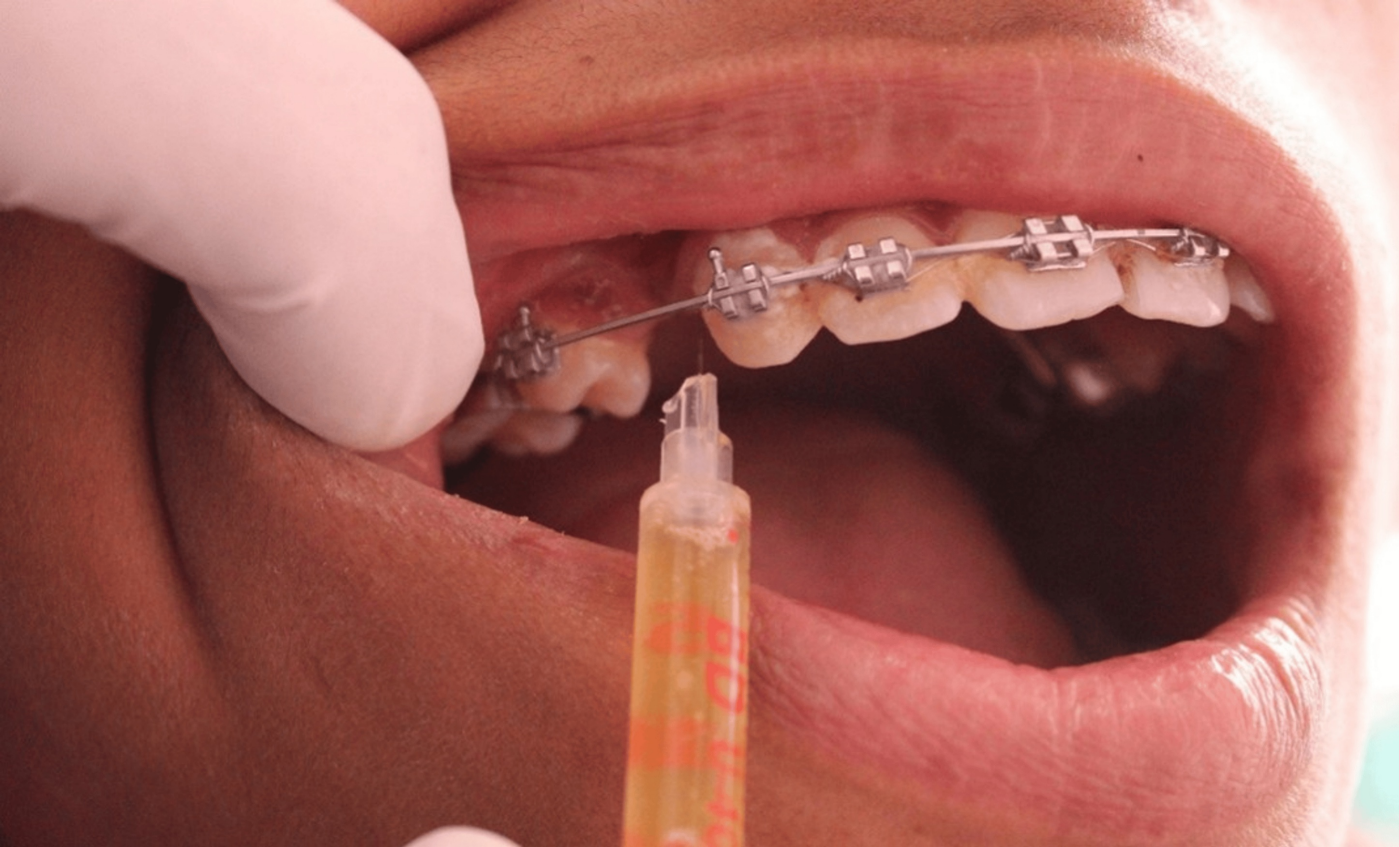
PRP injection at the intervention site PRP: Platelet-rich plasma

**Figure 3:**
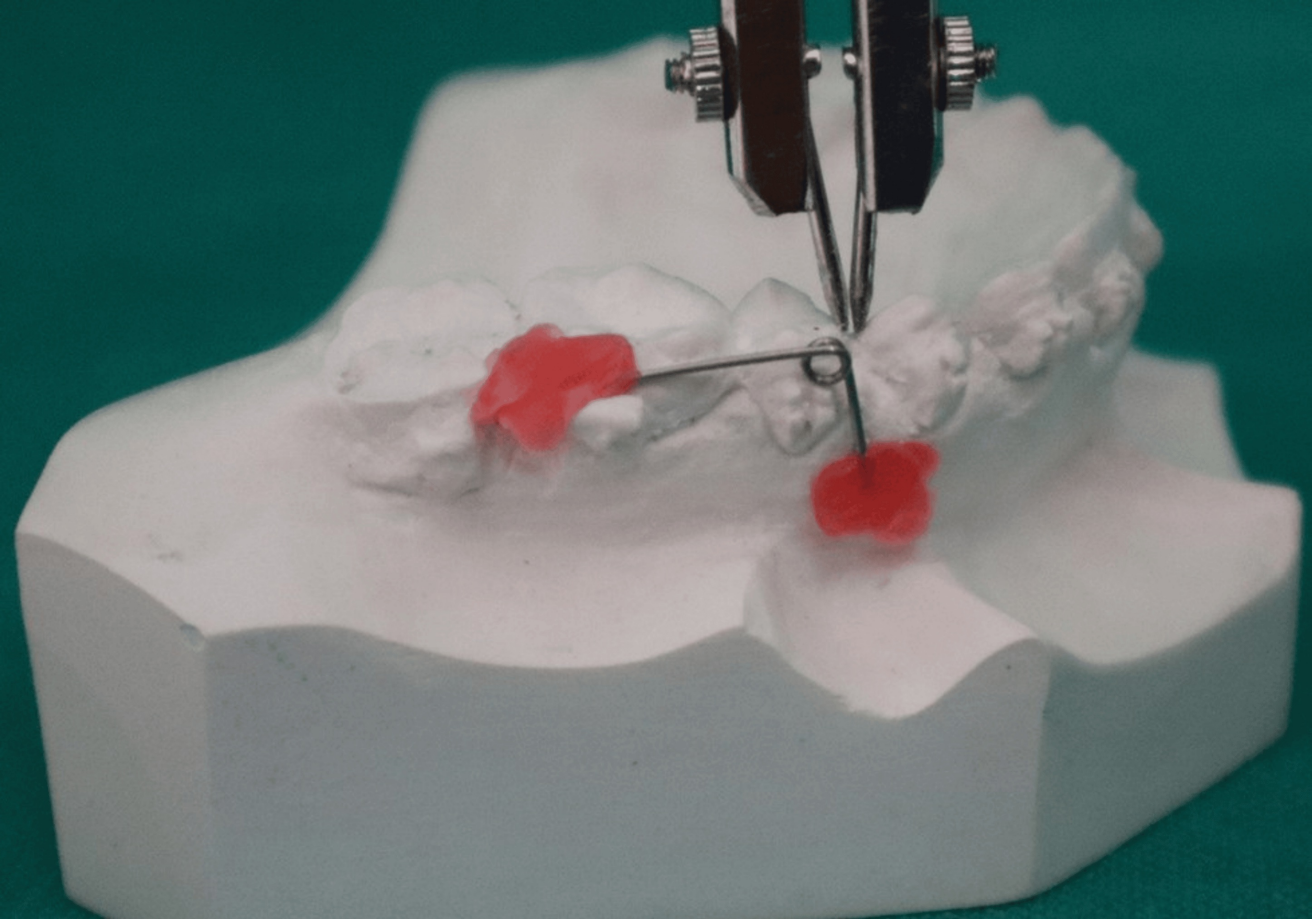
Measurement of canine retraction after completion of canine retraction done with the help of a Vernier caliper.

**Figure 4:**
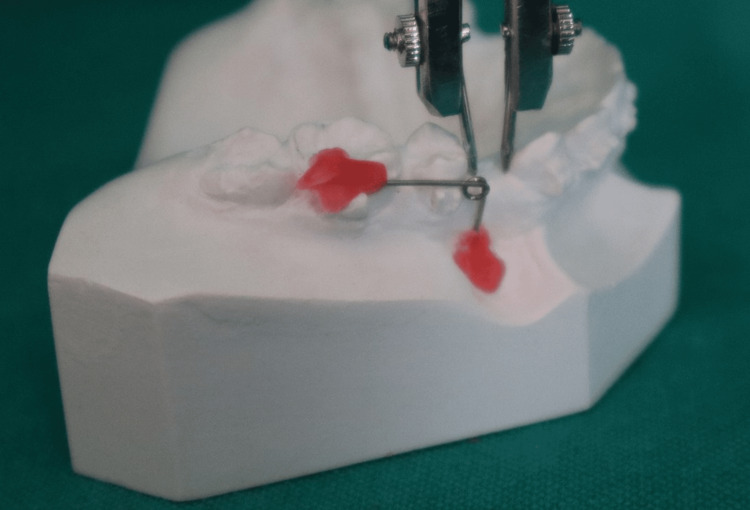
Measurement of space for canine retraction before the start of retraction with the help of a Vernier caliper.

The site was prepared for receiving PRP injection with 2% Local anaesthesia and was left for 10 mins. Then, 25 units (0.25 mL) of PRP were injected intraligamentary on the buccal side of the canine in the areas marked as mesiobuccal, middle, and distobuccal and on the palatal side, mesiopalatal and disto-palatal areas (5 units each area) together as depicted in Figure 2, showing PRP injected at the site. The intervention side was injected at the following intervals: 0, 21, and 42 days. Study impressions were taken before the start and after the end of the retraction procedure, as shown in Figures 3, 4.

Then, canine retraction was carried out on 0.019” × 0.025” SS wires using a closed coil spring with a constant force of 150 g measured with a Dontrix gauge by the same operator. Manual calliper measurements accurate to ± 0.001 mm were used to record on the 1st and 42nd day. This was done by means of a vernier calliper available in the department. Retraction amount was measured from the distal surface of the canine, and as a stable landmark canine eminence (canine fossa) was considered as the starting point. The distance between the contact points of the maxillary canine and second premolar was measured on both sides. Outcome measurements were performed by an independent examiner blinded to allocation status.

The readings were then subjected to statistical analysis. To address intra-examiner reliability, 10 study models were remeasured after a two-week interval. Intra-examiner reliability was assessed using the intraclass correlation coefficient (ICC) and Dahlberg’s formula for method error.

The study was powered to detect the predefined clinically meaningful difference between sides under the split-mouth superiority framework.

The 21-day endpoint was analyzed as a secondary interim timepoint to evaluate early acceleration effects.

Since this was a split-mouth design, the data are paired in nature. The statistical analysis has now been revised to appropriately reflect within-subject comparisons: Paired t-test (or Wilcoxon signed-rank test where applicable) was used for inter-side comparisons. Repeated-measures analysis was applied for time-dependent comparisons. Effect sizes with 95% confidence intervals were added to improve clinical interpretability.

Statistical analysis

Data obtained was coded and entered into Microsoft Excel 2007/2013. Descriptive and frequency analyses were done using Statistical Product and Service Solution (SPSS) (v.21.0) software. The data were found to be not normally distributed, as the p-value obtained from the Kolmogorov-Smirnov test was 0.032 (p < 0.05). 

## Results

The rate of canine retraction was measured in millimeters. Table 1 shows mean values for the rate of canine retraction in both groups (mm). The mean value of canine retraction at 0 day was 5.50±0.54 in the control group, with minimum and maximum values of 5.00 and 6.00, respectively. The mean at 21 days for the control group was 3.75±0.93 with minimum and maximum values of 3.00 and 5.50, respectively. At 42 days, the mean was 1.75±0.27 with 1.50 and 2.00 as minimum and maximum values, respectively. In the PRP group at 0 day, the mean value of canine retraction was 5.66±0.51 with minimum and maximum values of 5.00 and 6.00, respectively. At 21 days, the mean was 4.50±0.54 with 4.00 and 5.00 as minimum and maximum values, respectively. The mean at 42 days for the PRP group was 3.16±0.40 with minimum and maximum values of 3.00 and 4.00, respectively.

**Table 1:** Mean values for the rate of canine retraction in both groups (mm).

Group		Mean	SD	Min	Max
Control Group	0 day	5.50	0.54	5.00	6.00
	21 days	3.75	0.93	3.00	5.50
	42 days	1.75	0.27	1.50	2.00
PRP Group	0 day	5.66	0.51	5.00	6.00
	21 days	4.50	0.54	4.00	5.00
	42 days	3.16	0.40	3.00	4.00

Table 2 shows a comparison of mean values for the rate of canine retraction in both groups (mm) by the Mann-Whitney U test. The mean of canine retraction at 0 day was 5.50±0.54 and 5.66±0.51 in the control group and PRP group, respectively, with SEM of 0.166 and 95% class interval of -0.595 to 0.261, and the difference between the two groups was not statistically significant (p=0.363). At 21 days, 3.75±0.93 and 4.50±0.54 were the mean values for the rate of canine retraction in group control and group PRP, respectively. The SEM was 0.281 with 95% confidence interval of -1.47 to -0.026, and the difference between the groups was statistically significant (p=0.045). The mean canine retraction rate was 1.75±0.27 mm and 3.16±0.40 mm in the control group and PRP group, respectively, with SEM of 0.105 and 95% class interval of -1.937 to -1.395, and the difference between the two groups was not statistically highly significant (p<0.001). 

**Table 2:** Comparison of mean values for the rate of canine retraction in both groups (mm) (using the Mann–Whitney U test).

Time	Group	Mean±SD	SEM	95% CI		U value	p-value
				Lower	Upper		
0 day	Control Group	5.50±0.54	0.166	-0.595	0.261	-1.000	0.363
	PRP Group	5.66±0.51					
21 days	Control Group	3.75±0.93	0.281	-1.47	-0.026	-2.666	0.045*
	PRP Group	4.50±0.54					
42 days	Control Group	1.75±0.27	0.153	-1.811	-1.021	-9.220	<0.001**

Table 3 shows the comparison of mean values for the rate of canine retraction between the control and PRP groups performed using the Wilcoxon signed rank test. At baseline (0 day), the mean rate of canine retraction was 5.50 ± 0.54 mm in the control group and 5.66 ± 0.51 mm in the PRP group. The difference was statistically non-significant (Z = −1.000, p = 0.317), with four positive ranks, no negative ranks, and 26 ties. At 21 days, the mean rate of canine retraction reduced to 3.75 ± 0.93 mm in the control group, while the PRP group demonstrated a higher mean value of 4.50 ± 0.54 mm. However, the intergroup difference did not reach statistical significance (Z = −1.913, p = 0.056). Rank analysis showed 26 positive ranks and foyr negative ranks, with no ties. At 42 days, the mean rate further decreased to 1.75 ± 0.27 mm in the control group, whereas the PRP group showed a markedly higher mean value of 3.16 ± 0.40 mm. This difference was statistically significant (Z = −2.271, p = 0.023). All observations demonstrated positive ranks (n = 30) with no negative ranks or ties, indicating a significantly greater rate of canine retraction in the PRP group at this time point.

**Table 3:** Comparison of mean values for the rate of canine retraction in both groups (mm) (using the Wilcoxon signed rank test).

Time	Group	Mean±SD	Negative ranks	Positive ranks	Ties	Z value	p-value
0 day	Control Group	5.50±0.54	0	4	26	-1.000	0.317
	PRP Group	5.66±0.51					
21 days	Control Group	3.75±0.93	4	26	0	-1.913	0.056
	PRP Group	4.50±0.54					
42 days	Control Group	1.75±0.27	0	30	0	-2.271	0.023*
	PRP Group	3.16±0.40					

The intragroup comparison of mean canine retraction rates at different time intervals within the control group was analyzed using the Friedman test, followed by pairwise Wilcoxon signed rank tests as shown in Table 4.. The mean rate of canine retraction progressively decreased from 5.50 ± 0.54 mm at baseline (0 day) to 3.75 ± 0.93 mm at 21 days and further to 1.75 ± 0.27 mm at 42 days. The overall comparison showed a statistically significant difference across time intervals (χ² = 12.00, p = 0.002). Pairwise analysis revealed statistically significant reductions between all time points. The comparison between 0 day and 21 days demonstrated a significant decrease (Z = −2.214, p = 0.027), with all observations showing negative ranks (n = 30). Similarly, the reduction from 0 day to 42 days was statistically significant (Z = −2.220, p = 0.026), again with all observations showing negative ranks. The comparison between 21 days and 42 days also showed a significant reduction (Z = −2.214, p = 0.027), with all observations demonstrating negative ranks and no ties.

**Table 4:** Comparison of mean values for the rate of canine retraction at different time periods in the control group (mm) (using the Friedman test followed by the pairwise Wilcoxon signed rank test).

Time	Mean± SD	χ^2^ value, p-value	Comparison between	Negative ranks	Positive ranks	Ties	Z value, p-value
0 day	5.50±0.54	χ^2^=12.00 p=0.002*	0 day vs 21 days	30	0	0	-2.214, 0.027*
21 days	3.75±0.93		0 day vs 42 days	30	0	0	-2.220 0.026*
42 days	1.750±0.27		21 days vs 42 days	30	0	0	-2.214, 0.027*

Table 5 shows the intragroup comparison of mean values for the rate of canine retraction at different time intervals within the PRP group analyzed using the Friedman test followed by pairwise Wilcoxon signed rank tests. The mean rate of canine retraction showed a progressive decline from 5.66 ± 0.51 mm at baseline (0 day) to 4.50 ± 0.54 mm at 21 days and further to 3.16 ± 0.40 mm at 42 days. The overall comparison revealed a statistically significant difference across the three time intervals (χ² = 11.565, p = 0.003). On pairwise analysis, the comparison between 0 day and 21 days demonstrated a statistically significant reduction (Z = −2.070, p = 0.038), with 26 negative ranks and 4 ties. The reduction from 0 day to 42 days was also statistically significant (Z = −2.251, p = 0.024), with all 30 observations showing negative ranks and no ties. Similarly, the comparison between 21 days and 42 days revealed a statistically significant decrease (Z = −2.271, p = 0.023), with all observations demonstrating negative ranks and no ties.

**Table 5:** Comparison of mean values for the rate of canine retraction at different time periods in the PRP group (mm) (using the Friedman test followed by the pairwise Wilcoxon signed rank test). PRP: Platelet-rich plasma

Time	Mean± SD	χ^2^ value, p value	Comparison between	Negative ranks	Positive ranks	Ties	Z value, p-value
0 day	5.66±0.51	χ^2^=11.565 p=0.003*	0 day vs 21 days	26	0	4	-2.070, 0.038*
21 days	4.50±0.54		0 day vs 42 days	30	0	0	-2.251 0.024*
42 days	3.16±0.40		21 days vs 42 days	30	0	0	-2.271, 0.023*

## Discussion

One of the major lag factors for patients undergoing orthodontic treatment is the increased duration of treatment time, which makes one question whether to commence the treatment or not.

To overcome this failure of orthodontic treatment, various research was undertaken to increase orthodontic tooth movement. Accelerated Orthodontics is considered a boon for decreasing the treatment time and thereby increasing the patient satisfaction index [24-27]. Literature reports reveal various methods of accelerated orthodontics that include invasive and non-invasive methods. One of these is a minimally invasive procedure that includes the administration of PRP at the site to bring about accelerated tooth movement in the area of concern. To reassess the effect of PRP in accelerated tooth movement, this prospective, split-mouth study was undertaken.

The data of the present trial showed a higher rate of canine retraction in the study side of the trial when compared to the control side, with a total retraction of the canine of 2.5mm and 3.8 mm for the control and the study sides, respectively. The results were statistically significant (p =0.022). The results of the present study are in agreement with Gulec et al. [28], who recorded that PRP accelerates OTM. They further stated that PRP injections increase OTM by 1.4 to 1.7 times, and PRP can be beneficial in patients who are undergoing a long treatment procedure because of the underlying malocclusive trait.

A similar study was undertaken by Rashid et al. [17] wherein they injected PRP in a dog sample and evaluated the rate of retraction. He further concluded his study by stating that injecting PRP would accelerate tooth movement without having any side effects. Concurrent results were found in a study conducted by El Gazzar et al. [29]. In a study undertaken by Arora et al. [30], dissimilar results were found wherein they injected 4 folds of PRP in the study side and the other quadrant as the control side, but concluded that even 4 folds of PRP injections could not accelerate the orthodontic tooth movement. Abrar et al. [31] and Ammar et al. [8] conducted studies with similar results to our study, which also concluded with the accelerating effect of PRP on the rate of retraction. Concurrent results were found by Liou et al. [15], who stated that PRP would accelerate tooth movement by 1.7-fold in the intervention site.

When compared to other methods of canine retraction, minimally invasive procedures [26,31-33] showed 1.18 (± 0.04) mm on the intervention side, while PRP showed canine retraction of 2.5mm on the intervention side, showing canine retraction was faster in the PRP group as compared to the laser group. Similar to the acceleration reported with PRP in the present study, Alam et al. [34] and Alfawal et al. [35] demonstrated enhanced orthodontic tooth movement with low-level laser therapy, suggesting that biologically mediated, minimally invasive approaches may provide comparable short-term benefits. While biologically driven acceleration techniques are considered minimally invasive, careful monitoring remains essential. A recent systematic review by Sirri et al. [36] emphasized the importance of evaluating potential adverse effects, including root resorption, when employing acceleration strategies. Although PRP is autologous and biologically favorable, long-term radiographic evaluation is necessary to confirm its safety profile. As a minimally invasive procedure, PRP can be used as a potential adjunct to accelerate tooth movement in day-to-day practice.

Limitations

Despite the promising short-term findings, certain limitations must be acknowledged. The observation period was limited to 42 days, which restricts conclusions regarding long-term acceleration patterns and post-retraction stability. Limitations also include a relatively small sample size and a lack of detailed power justification for each time point, as well as short follow‑up for root resorption assessment, and potential contralateral biological cross‑effect inherent to split‑mouth designs. Additionally, long-term outcomes such as relapse tendency and sustained periodontal health were not evaluated. Future longitudinal studies with extended follow-up are required to determine the durability and clinical significance of PRP-mediated acceleration.

## Conclusions

The present study concluded that PRP injections could accelerate the amount of tooth movement faster in the intervention site than in the control group. Hence, PRP can be used to accelerate orthodontic tooth movement. PRP is beneficial with its major advantage being a minimally invasive procedure rather than the other methods of acceleration that are majorly invasive and also does not have any systemic major side effects. Hence, PRP can be useful for decreasing the treatment time of orthodontic patients. PRP may represent a minimally invasive adjunctive approach for short-term acceleration of orthodontic tooth movement, but further randomized controlled trials with extended follow-up time are necessary before definitive clinical recommendations can be made.
